# P06-12 Construction and validation of a physical activity and sedentary behavior temperaments questionnaire among French adults

**DOI:** 10.1093/eurpub/ckac095.097

**Published:** 2022-08-29

**Authors:** Florian Manneville, Laurent Muller, Claudia Collmann, Laure Truwant, Abdou Yacoubou Omorou

**Affiliations:** 1 APEMAC, Université de Lorraine, Vandoeuvre-Lès-Nancy, France; 2 CIC-1433 Clinical Epidemiology, CHRU de Nancy, Vandoeuvre-Lès-Nancy, France; 3 APEMAC, Université de Lorraine, Metz, France; 4 Université de Lorraine, Metz, France; 5 CIC-1433 Clinical Epidemiology, CHRU de NANCY, Vandoeuvre-Lès-Nancy, France

**Keywords:** Psychometric validation, Temperament, Physical activity, Sedentary behavior

## Abstract

**Background:**

Temperament refers to innate differences between individuals, is partly genetically determined, relatively stable across lifespan and expressed through behaviors such as physical activity and sedentary behavior. These two behaviors are known as major determinants of health. Therefore, measuring physical activity and sedentary behaviors temperaments appears to be of interest but no existing questionnaires allow for it among French adults. This study aimed to create and validate a questionnaire to measure physical activity and sedentary behavior temperaments among French adults.

**Methods:**

The questionnaire was created by the Delphi method. Based on an existing questionnaire on eating temperament, 31 experts in physical activity, health psychology and public health were asked to formulate equivalent items to measure physical activity and sedentary behavior temperaments. The test of the psychometric qualities of the questionnaire and its validation will be carried out on three samples of north-eastern French adults: one of 500 to explore internal validity, one of 100 for external validity, and one of 60 for test-retest reliability (4-week interval). Internal validity will be investigated by exploratory and confirmatory factor analyses, and external validity and test-retest reliability with correlation analyses.

**Results:**

The Delphi method results in a questionnaire of 40 items on physical activity and sedentary behavior temperaments. The test of the psychometric qualities of the questionnaire and its validation are in progress and will be carried out for the congress.

**Conclusions:**

The validation of this questionnaire and its use in practice would help to guide changes in the management of physical activity and sedentary behavior as part of health promotion approach.

